# Deep reinforcement learning-based multi-lane mixed traffic ramp merging strategy

**DOI:** 10.1371/journal.pone.0331986

**Published:** 2025-09-18

**Authors:** Tong Zhou, Yuzhao Huang, Yudan Tian, Hua Huang, Minghui Ou, Tao Lin

**Affiliations:** 1 College of Big Data and Internet of Things, Chongqing Vocational Institute of Engineering, Chongqing, China; 2 International Division, Chongqing Vocational Institute of Engineering, Chongqing, China; 3 School of Electronics and Internet of Things, Chongqing Polytechnic University of Electronic Technology, Chongqing, China; Southwest Jiaotong University, CHINA

## Abstract

Due to concentrated conflicts, on-ramp merging is an important scenario in the study of new hybrid traffic control. Current research mainly focuses on optimizing the vehicle passage sequence of ramp vehicles merging with mainline vehicles in single-lane scenarios, neglecting the coordination problem of vehicles in multiple mainline lanes. Therefore, an Improved Dueling Double DQN (D3QN) On-ramp Merging Strategy (IDS stands for the initials of Improved, D3QN, and Strategy) combined with a sine function is proposed, establishing a Vehicle Coordination System (VCS) to guide the merging of vehicles in multi-lane mainline traffic. This strategy uses the improved D3QN algorithm combined with the excellent smoothness of the sine function to evaluate driving safety, helping vehicles find suitable gaps in traffic flow. An action masking mechanism was deployed during the strategy exploration phase to prevent unsafe actions. The proposed VCS + IDS strategy was tested in SUMO simulations of on-ramp merging under different density of vehicle flow. Under a traffic flow of 1200 vehicles per lane per hour, the on-ramp merging completion rate of VCS + IDS reached 98.62%, and the task completion rate was 98.11%, which increased by 11.08% and 10.79% compared to traditional D3QN, respectively, validating the effectiveness of this method.

## 1. Introduction

On-ramp merging is a high-risk road traffic scene that requires merging with a safe speed within a limited distance due to the compulsory nature of the on-ramp merge. The inability of autonomous vehicles to respond in a timely manner to dynamic driving environments, especially in mixed traffic with human-driven vehicles, makes it one of the most challenging decision-making scenarios for autonomous vehicles [1,2]. The task of autonomous vehicle ramp entry is currently mainly addressed using two methods, including mathematical models and deep reinforcement learning, with mathematical model methods consisting of optimization-based and rule-based approaches [3].

Optimization-based ramp merging control methods mainly using optimization algorithms to determine the optimal control strategy for autonomous vehicles during high traffic loads. A key step is to create a mathematical model that accurately describes the motion state of vehicles. For example, Cao et al. proposed a method for generating cooperative merging paths for autonomous vehicles in ramp merging scene using model predictive control. The results showed that cooperative merging paths could be successfully generated in various traffic scene without the need to readjust optimization parameters [4]. Rios-Torres and Malikopoulos proposed an optimization framework and provided a closed-form analytical solution to enable smooth traffic flow without interruption in the merging area [5]. Zhou et al. proposed a vehicle trajectory planning method for automated ramp merging, expressing it as two related optimal control problems. To find the best solution, the Pontryagin Maximum Principle (PMP) was used to provide optimal solutions for the trajectories of vehicles merging from the ramp and those on the mainline [6].

Rule-based on-ramp merging methods rely on a set of pre-determined rules to address the issue. These methods are easy to implement and widely adopted, but defining the rules can be challenging. For instance, Ding proposed a rule-based cooperative merging adjustment algorithm for connected vehicles in the ramp area. The algorithm includes a central controller that assigns vehicle arrival times and determines the optimal merging sequence for mainline and ramp vehicles, reducing travel time in the merging area [7]. In the Vehicle-to-Vehicle (V2V) environment, Shi proposed a rule-based vehicle cooperative merging model to complete ramp merging. By selecting suitable gaps and establishing a linear time-discrete model, vehicle trajectories are optimized to achieve merging [8]. Hu and Sun proposed an online system control algorithm suitable for multi-lane highway merging areas. The algorithm coordinates traffic flow within the merging area by optimizing vehicle lane-changing and following trajectories. It adjusts lane traffic upstream of the merging point using rule-based lane-changing decisions, thereby balancing the traffic distribution in the downstream lanes [9].

Optimization-based on-ramp merging methods aim to improve road traffic efficiency and safety by optimizing vehicle control strategies. However, it is important to note that different optimization algorithms must be customized for specific traffic environments and vehicle densities. Moreover, the generalizability of the algorithms may be limited. These rule-based models have limitations in terms of agent control due to the difficulty in defining rules and constraints on discrete actions. In contrast, learning-based methods can automatically learn the relationship between continuous control inputs and outputs, addressing the limitations of rule-based methods [10]. Therefore, in recent years, learning-based methods have garnered increasing attention and development.

With the advancement of machine learning technologies in recent years, sophisticated machine learning algorithms have been widely applied in the development of autonomous driving vehicles [11,12]. However, these methods are mostly data-driven, requiring a large amount of offline driving data (usually with labeled tags) to cover all possible scenarios [13]. This heavy reliance on extensive training data limits the application of machine learning methods in autonomous driving, as it is challenging to collect large-scale datasets containing a variety of scene, and the labeling work is time-consuming [14]. In contrast, deep reinforcement learning offers a solution to these limitations, as the data for DRL training can be obtained from the interaction between the agent and the environment, and the training process does not require labeled tags. This not only reduces the dependence on large-scale labeled datasets but also allows for trial-and-error learning in various driving scenarios [15,16]. In the application of DRL algorithms, if an agent takes an incorrect action, it will be punished to reduce the likelihood of repeating that action in the same state. For the solution to on-ramp merging, Li et al. proposed a new safety indicator—time difference to merging (TDTM), combined with the classic time to collision (TTC) indicator to evaluate driving safety, assisting merging vehicles in finding suitable gaps in the traffic flow, thereby improving driving safety [17]. Chen et al. developed an effective reinforcement learning method, integrating action masks, curriculum learning, and parameter sharing. Experimental results show that the proposed method outperforms existing methods in both training efficiency and collision rate [18]. Zhang et al. proposed an IPPO method based on proximal policy optimization algorithm, which is based on autonomous learning and parameter sharing strategy, establishing an autonomous driving behavior decision model [19].

The aforementioned centralized control methods mainly consider the case where there is only one lane on the mainline, or assume that vehicles on the mainline do not change lanes. However, in real on-ramp merging traffic scenarios, the mainline usually has two or more lanes, and if the flow in other lanes is smaller, vehicles on the mainline often choose to enter the lane with less traffic in advance to avoid conflicts with ramp vehicles. Some scholars have studied the ramp merging problem in multi-lane scenarios. Hou et al. proposed a hierarchical model of collaborative on-ramp merging control (CORMC) for mixed traffic, where the upper layer uses a predictive position search (APS) algorithm to predict the merging location, and the lower layer uses a cooperative merging control (CMC) model to ensure the safety and smooth execution of the merging vehicle [20]. This algorithm only considers the elimination of conflicts between a single ramp vehicle and mainline vehicles in multi-lane scenarios, without considering the unbalanced lane traffic caused by a large number of ramp vehicles merging into the mainline. Most current research focuses on the assumption that fully autonomous driving vehicles (CAVs) will be widely adopted, which is overly idealistic. In reality, full-scale adoption may still take some time. During this transitional period, the coexistence of Connected and Autonomous Vehicles (CAVs) and Human-Driven Vehicles (HDVs) in traffic flows will become the norm, increasing the complexity of the traffic environment. However, research on this mixed traffic condition is still relatively scarce

Therefore, this paper designs a deep reinforcement learning-based multi-lane mixed traffic strategy for on-ramp merging, with the main contributions including,

(1) A novel Vehicle Coordination System (VCS) is established, which is embedded in roadside units and receives real-time information through Vehicle-to-Vehicle (V2V) communication. It balances the traffic flow between different lanes in real-time, optimizes the use of multiple lanes, and addresses the issue of uneven traffic flow across lanes.
(2) A novel D3QN algorithm improved with sine functions is proposed for on-ramp merging strategies, utilizing the smoothness of sine functions for horizontal control in lane-changing strategies. Longitudinal control employs the car-following model of the Intelligent Driver Model (IDM), assisting vehicles in finding suitable gaps within the traffic flow.
(3) An action shielding mechanism is proposed to ensure that post-merge actions are safe. Since agents based on DRL learn through trial and error, they may take actions that threaten traffic safety during the strategy exploration phase. To address this issue, an action shielding mechanism is incorporated after the ramp vehicle reaches the merging point, ensuring driving safety in various on-ramp merging scenarios.

The rest of the paper is organized as follows. Section II introduces the division of the on-ramp merging scenario area and the operating mechanism of the Vehicle Coordination System. Section III introduces the proposed IDS Ramp Merging Strategy. Section IV analyzes and discusses the simulation results. Section V discusses the proposed method on the basis of the results and concludes the study.

## 2. Vehicle coordination system framework

**Fig 1:**
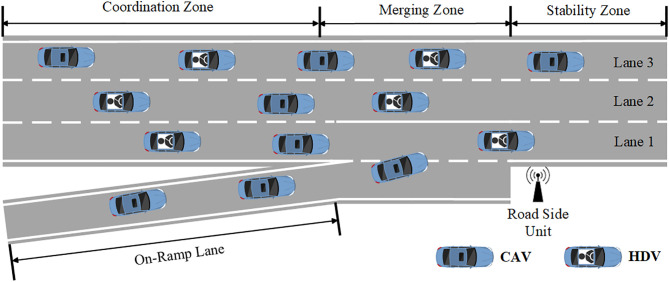
Zone division of On-ramp merging scenario.

The inflow of ramp vehicles often leads to a sharp increase in the traffic density of the mainline lanes adjacent to the ramp [21,22]. Due to the need to maintain a safe distance between vehicles, following vehicles typically slow down to avoid collisions [23], ultimately leading to traffic congestion. The on-ramp merging scenario studied in this paper involves an urban expressway with three mainline lanes and one ramp, as shown in **Fig 1**. The section is divided into three areas: the coordination area, the merging area, and the stabilization area. Marked with CAV represent Connected Automated Vehicles, and CHV represent Connected Human-Driven vehicles. This paper proposes an efficient Vehicle Coordination System (VCS) for coordinating vehicles in multi-lane traffic. For the entire VCS system, the following assumptions are made,

(1) The VCS is embedded within the Road side Unit (RSU). All vehicles on all lanes are communicative vehicles capable of exchanging real-time status information through Vehicle-to-Vehicle (V2V) communication without considering communication latency.
(2) Assuming that the behavior of CAVs (Connected and Autonomous Vehicles) is fully controllable and deterministic, while the behavior of HDVs (Human-Driven Vehicles) is uncontrollable and follows the IDM (Intelligent Driver Model) car-following model, which limits their ability to perform spontaneous lane changes. This simulates obstacles in traffic that prompt CAVs to change lanes to alleviate road traffic congestion.

In the architecture of the Vehicle Coordination System, each vehicle is controlled by its own controller, achieving multi-vehicle cooperative control through information exchange between vehicles. Within the coordination area, VCS can globally perceive the average density of each lane, guiding CAVs and HDVs to choose lanes with lower traffic volumes through lane-changing strategies. Fully utilizing multiple lanes can alleviate the issue of sudden increases in traffic density between lanes and traffic flow saturation. In VCS, all autonomous vehicles can wirelessly communicate with VCS and ignore the impact of communication delays. Each CAV entering the coordination area has a unique identifier. When a vehicle enters the coordination area, it begins communicating with VCS. The vehicle transmits its status information to VCS and receives global lane status information from VCS. Lane-changing strategies make the best choices between maintaining longitudinal motion and lane-changing strategies based on the vehicle’s status information. In the merging area, vehicles on the ramp search for safe merging gaps in the first lane. Through information exchange with VCS, ramp vehicles cooperate with adjacent vehicles in the first lane of the merging area to reserve a safe merging interval. Since the merging behavior of ramp vehicles is extremely similar to lane-changing behavior, the merging of ramp vehicles also uses the lane-changing decision model. Ramp vehicles execute the merging operation, reach the expected position, and safely exit the stable area to complete the task.

## 3. IDS ramp merging strategy

### 3.1. Lateral control combined with sine function

During the lane-changing process in the coordination area, a vehicle’s lane change (LC) is decomposed into two independent movements: lateral and longitudinal. Laterally, the designed lane-changing trajectory takes into account the safety and comfort of driving. A sine function curve is used to determine the trajectory [24], which has the following two main advantages and ensures the smoothness of the continuous second derivative of the lane-changing trajectory.

**Fig 2:**
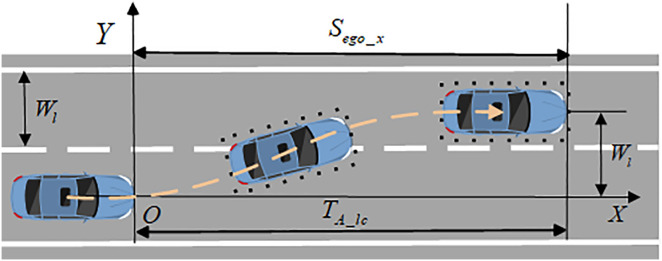
Vehicle lane change trajectory.


The specific construction process of the LC trajectory is as **Fig 2** shows, where $S_{ego\_x}$ is the longitudinal travel distance during the vehicle’s lane change process, and $W_{l}$ is the lane width. Based on the shape, speed, and acceleration characteristics of the lane-changing trajectory, the longitudinal and lateral axes of the lane are taken as the X and Y coordinates, respectively, with O as the starting point of the lane change, to establish a lane-changing trajectory model based on the sine function. The mathematical description equation of the lane-changing trajectory $S_{\left(x\right)}$ is as follows:

$$S(x)=\frac{W_{l}}{2\pi }\left[\frac{2\pi }{S_{ego\_x}}x-\sin\left(\frac{2\pi }{S_{ego\_x}}x\right)\right],0\leq x\leq S_{ego\_x}$$ (1)

Based on the vehicle kinematic equations, the following expressions can be derived:


$$\{\begin{array}{c} {}*20cS_{ego\_x}=\overset{―}{\nu_{ego\_x}}T_{A\_lc}\\ x=\overset{―}{\nu_{ego\_x}}t \end{array},0\leq t\leq T_{A\_lc}$$ (2)

where $T_{A\_lc}$ is the total duration of the coordinated lane-changing process, and $\nu_{ego\_x}$ is the average longitudinal velocity during the coordinated lane-changing process. Combining Eq. (1) and (2), the trajectory equation $y_{ego}(t)$ of the ego vehicle during the lane-changing phase can be obtained,


$$y_{ego}(t)=\frac{W_{l}}{2\pi }\left[\frac{2\pi }{T_{A\_lc}}t-\sin\left(\frac{2\pi }{T_{A\_lc}}t\right)\right]$$ (3)

By calculating the first and second derivatives of Eq. (3) respectively, the expressions for the lateral velocity $v_{ego\_y}(t)$ and acceleration $a_{ego\_y}(t)$ during the lane change can be obtained,


$$\nu_{ego\_y}(t)=\frac{W_{l}}{2\pi }\left[\frac{2\pi }{T_{A\_lc}}-\frac{2\pi }{T_{A\_lc}}\cos\left(\frac{2\pi }{T_{A\_lc}}t\right)\right]$$ (4)

$$\begin{array}{c} a_{ego\_y}(t)=\frac{2W_{l}\pi }{{T_{A\_lc}}^{2}}\sin\left(\frac{2\pi }{T_{A\_lc}}t\right) \end{array}$$ (5)

Based on Eq. (5), it can be inferred that the maximum lateral acceleration of the ego vehicle occurs at time $t=T_{A\_lc}/4$, and the maximum lateral acceleration $a_{y\max}$ is,


$$a_{ymax}=\frac{2W_{l}\pi }{{T_{A\_lc}}^{2}}$$ (6)

Integrating Eq. (6) into Eq. (5) and (3), allows the lateral trajectory and acceleration during the LC to be described as the expressions that include the terms for the maximum lateral acceleration $\left(a_{y\max}\right)$ and the coordinated LC duration $\left(T_{A\_lc}\right)$,


$$y_{ego}(t)=\frac{a_{ymax}T_{A\_lc}}{2\pi }t-\frac{a_{ymax}{T_{A\_lc}}^{2}}{4\pi^{2}}sin\frac{2\pi t}{T_{A\_lc}}$$ (7)

$$a_{ego\_y}(t)=a_{ymax}sin\frac{2\pi t}{T_{A\_lc}}$$ (8)

Therefore, throughout the entire process in the coordination area, the lateral trajectory and acceleration of the ego vehicle during LC are as shown in Eq. (7) and (8). Since the ego vehicle changes lanes from the centerline of the original lane to the centerline of the target lane, its longitudinal displacement is exactly equal to the width of the lane. According to the road construction industry standards in China, the lane width is set to 3.75 meters in this paper. According to the sine function curve law, the ego vehicle moves to the boundary of the two lanes at time $t=T_{A}/2$, and this time is considered as the moment when the ego vehicle just leaves the original lane and begins to enter the target lane.

### 3.2. Markov decision process model

**Fig 3:**
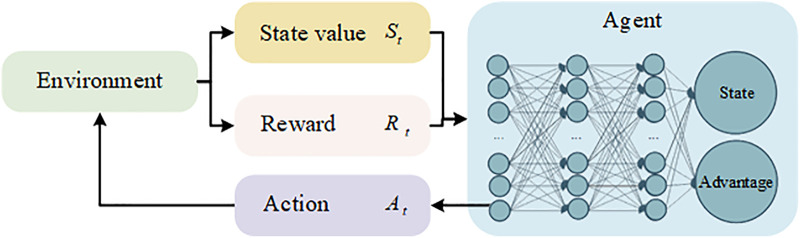
MDP model for behavioral decision making in autonomous vehicles.

Deep reinforcement learning algorithms are applied to solve sequential decision-making problems, which can be modeled within the framework of a Markov Decision Process (MDP). Research has shown that decision-making problems in autonomous driving can be regarded as an MDP problem [25]. MDP is a mathematical model for decision-making defined by the tuple (S,A,T,R,γ), where S is the state space, A is the action space, T describes the transition probabilities between states, R is the reward function, and γ∈[0,1] is the discount factor. In this model, an agent selects an action based on the current state of the environment, and the environment transitions to the next state as a result of the executed action. Then, the agent receives a scalar reward defined by the reward function R. This process is repeated until the end of each episode. The goal of the DRL model is to learn an optimal policy $\pi^{*}$ that maps states to actions, maximizing the sum of future discounted rewards. The cumulative reward G is calculated as follows,


$$\begin{array}{c} G=E[\sum_{k=0}^{\infty }\gamma^{k}r_{t+k+1}] \end{array}$$ (9)

where E denotes the mathematical expectation, and k is the k-th time point after the current time t. This study aims to use the DRL model to solve the decision-making problem of autonomous driving in multi-lane mixed traffic scene at on-ramp merging. In conjunction with CSAT, the D3QN algorithm is used to train vehicles to successfully merge into the main road, to increasing the overall traffic volume of the road section. Vehicles interact with the environment and receive numerical rewards, which are used to learn the optimal driving strategy to maximize the expected return of the current state, as shown in **Fig 3**. The DRL method solves the decision-making problem by using the MDP framework. The model mainly consists of the state space 𝑆, action space 𝐴, and reward function 𝑅. The agent controls the vehicle, and during the driving process, it obtains the current road environmental state information $S_{t}$ from the VCS (where t represents the number of steps in the current round), and then selects and executes actions $A_{t}$ according to the environmental state and its policy. The environment provides feedback to the vehicle in the form of rewards $R_{t}$ based on the current state and the vehicle’s motion, allowing the vehicle to improve its action strategy, and the environment transitions to the next state $S_{t+1}$. The above iterative process is repeated until the system converges or reaches a preset number of iterations.

#### 3.2.1. State space.

**Fig 4:**
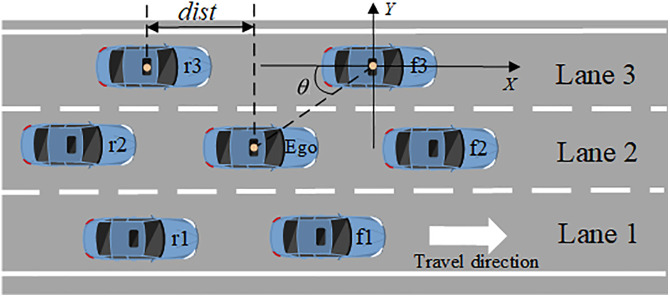
Relative distance from surrounding vehicles.


The state space refers to the information that a vehicle can perceive and obtain during its motion. It includes the position and motion state of the ego vehicle, as well as the state information of the surrounding traffic vehicles. The ego vehicle is equipped with a radar with a range of 125 meters, which can collect information such as the position and speed of vehicles within the perception range [26]. As described in **Fig 4**, it illustrates the relative positions of the ego vehicle and the surrounding vehicles. When making lane selection decisions, vehicles need to extract features from the surrounding environmental information as state inputs. The proposed method considers the six closest vehicles on the main road within the perception range during the development process, including the three vehicles in front of the ego vehicle (f1, f2, f3) and the three vehicles behind the ego vehicle along the direction of the traffic flow (r1, r2, r3). If there are no vehicles within the radar range, zero padding is added to the state space. The state of each main road vehicle is defined as follows,

$$S_{env}=\{S_{ego},S_{f1},S_{f2},S_{f3},S_{r1},S_{r2},S_{r3},tf_{1},tf_{2},tf_{3}\}$$ (10)

where $S_{ego}$ is the state information of the ego vehicle, $tf_{1},tf_{2},tf_{3}$ is the traffic density of each lane. $S_{f1},S_{f2},S_{f3},S_{r1},S_{r2}$, $S_{r3}$ is the state information of the surrounding vehicles. Each piece of information 𝑆 includes seven state features. Taking $S_{f1}$ as an example,


$$S_{f1}=\{isPresence,x,y,v_{x},v_{y},\cosh,\sinh\}$$ (11)

where isPresence indicates the presence or absence of a vehicle, x and y are the longitudinal and lateral positions of the vehicle, respectively. $v_{x}$ and $v_{y}$ are the longitudinal and lateral velocities of the vehicle. cosh and sinh are the cosine and sine values of the vehicle’s heading angle.

#### 3.2.2. Action space.

In this model, the action space refers to all possible driving behaviors that a vehicle can exhibit in the environment. The action space is designed as follows: the vehicle’s acceleration control input is set within the range of [−4.5, 2.6] $m/s^{2}$ [27]. To ensure the safe driving of the vehicle on the main road, an action shielding mechanism is proposed. If the distance between vehicles is $dist_{r}\leq 5$, an emergency braking action is triggered, setting the acceleration to acc=−4.5, and a negative reward is returned from the environment. It is defined as $dist_{r}$,


$$dist_{r}=\sqrt{{\left(x_{ego}-x_{r}\right)}^{2}+{\left(y_{ego}-y_{r}\right)}^{2}}-veh_{l}$$ (12)

where $veh_{l}$ is the length of the vehicle, assumed to be 5 meters.

The action set in the Markov Decision Process is:


$$A=(keep,lc_{l},lc_{r})$$ (13)

where keep is to stay in the current lane, $lc_{l}$ is to change lanes to the left, $lc_{r}$ is to change lanes to the right. When a decision is made that the vehicle needs to perform a LC action, the lateral trajectory from Eq. (7) and the acceleration from Eq. (8) are executed.

#### 3.2.3. Reward function.

Rewards are the responses of the environment to the actions of the agent, and the value of the reward is determined by the created reward function, which affects the effectiveness of the learning process and the achievement of expected results. In the on-ramp merging scene, to ensure the safe and efficient driving of vehicles on the road, the design of the reward function must take into account both driving efficiency and dangerous driving behaviors. This means that while rewarding efficient driving, dangerous driving must also be punished.

To enable the vehicle to learn safe driving while interacting with the environment, a safety reward function is designed. To teach the vehicle to avoid collisions, if it collides with other vehicles during lane changing and merging processes, then a penalty is applied, $R_{safe}=-100$.


$$R_{safe}=\{\begin{array}{cc} {}*20l-100, & collision\\ 1, & others \end{array}$$ (14)

To optimize the overall travel time and encourage the vehicle to maintain the best speed, the efficiency reward function is defined as follows:


$$R_{eff}=-\left|v_{\max}-v_{ego}\right|$$ (15)

where $v_{ego}$ is the current vehicle speed, and $v_{\max}=30m/s$ is the maximum expected speed, which is set according to the normal safe driving speed on highways in the real world. This reward provides a certain negative reward when the vehicle is driving too slowly or speeding, effectively avoiding unsafe driving behaviors.

Considering that frequent lane changes may reduce the overall traffic smoothness and increase safety risks, the LC reward function penalizes obviously unnecessary lane change behaviors. To encourage vehicles to choose the optimal lane and alleviate the fluctuations in the main traffic flow caused by the merging of ramp vehicles, the lane change reward function is defined as follows,


$$R_{lc}=\{\begin{array}{cc} {}*20l20\Delta tf, & \Delta tf<0\\ 10\Delta tf, & \Delta tf\geq 0 \end{array}$$ (16)

Δtf=σ(t−1)−σ(t) (17)

$$\sigma \left(t\right)=\frac{\sqrt{\frac{\text{1}}{\text{3}}\sum_{i=1}^{3}{\left(tf_{i}(t)-\frac{\text{1}}{\text{3}}\sum_{i=1}^{3}tf_{i}(t)\right)}^{2}}}{\frac{\text{1}}{\text{3}}\sum_{i=1}^{3}tf_{i}(t)}$$ (18)

where σ(t) is the overall traffic density at time t, and $tf_{i}$ is the traffic density of each lane. When a vehicle changes lanes and exacerbates the overall traffic density, it will receive a negative reward.

To assess the overall completion of the task, the successful merging of the ramp vehicle and its safe travel on the main road afterward are evaluated. If the ramp vehicle safely reaches the merging area, $R_{task}=60$. If it safely exits the stabilization area, $R_{task}=100$, and if it fails to reach or arrives at the destination after the timeout, $R_{task}=-100$.

Considering the above factors, the total reward function is defined as,


$$R=w_{safe}\times R_{safe}+w_{eff}\times R_{eff}+w_{lc}\times R_{lc}+w_{task}\times R_{task}$$ (19)

where 𝑤 is the weight for each reward component. After trial and error, the optimal values for $w_{safe}$, $w_{eff}$, $w_{lc}$, and $w_{task}$ are 0.1, 0.2, 0.1, and 0.05, respectively. In this study, the observation space, state space, and reward function are selected based on the optimal results from preliminary experiments, where different options were examined.

### 3.3. IDS algorithm

In reinforcement learning, it is necessary to estimate the value for each state, but for many states, it is not necessary to estimate the value for each action. The Dueling Double Deep Q-Network (D3QN) adds a Dueling Network Architecture(DNA) to both the main network and the target network on the basis of the Double Deep Q-Network (DDQN) [28]. The DNA separates the state value and the advantage of the state-action pair, allowing for a more accurate assessment of the Q-values. The uniform random sampling used in Deep Q-Networks (DQN) [29], DDQN, and Dueling DQN [30] algorithms is not optimal. During the interaction with the environment, the experience replay unit continuously stores experience samples for model training, and these data may be kept in the experience replay unit indefinitely. Frequent replay of these experiences can make the agent aware of correct or incorrect actions, thereby constantly correcting them. However, the importance of different experience samples varies. Since the samples in the experience replay unit are constantly being updated, if a small number of samples are taken uniformly at random from the experience replay unit as model input, some samples with higher importance may not be fully utilized and may even be directly overwritten, leading to reduced model training efficiency.

To improve the training efficiency of the model, this paper adopts prioritized experience replay [31], which increases the probability of extracting more important samples. It uses a proportional prioritization mechanism that defines the priority of experience,


$$p_{t}=|\delta_{t}|+\epsilon$$ (20)

where ϵ is a small constant number that ensures samples with a TD-error almost equal to zero also have a lower probability of being extracted.

Based on this, the probability of sampling experience t can be defined by Eq. (22),


$$P_{t}=\frac{p_{t}^{\alpha }}{\sum_{n}p_{n}^{\alpha }}$$ (21)

where n is the size of the replay experience unit. α∈[0,1] controls the degree of prioritization used. α=0 implies uniform sampling, while α=1 implies greedy strategy sampling.

Due to the frequent replay of experiences with high temporal difference (TD) errors and the overly frequent access to certain states, the lack of diversity in experience can lead to overfitting in network training. Therefore, importance sampling weights w can be used for correction, as shown in Eq. (23),


$$w_{i}=1/{\left(\frac{p_{i}}{p_{\min}}\right)}^{\beta }$$ (22)

where $p_{i}$ represents the probability of sampling experience i, $p_{\min}$ represents the minimum sampling probability, and the parameter β represents the degree of correction.

The loss function L(w) of the Q-network is defined in Eq. (24),


$$L(w)=\sum w(t)(y_{t}-Q(S_{t-1},A_{t-1};\theta ,\alpha ,\beta ){)}^{2}$$ (23)

where $y_{t}$ represents the target Q-value at time t, and $Q(S_{t-1},A_{t-1};\theta ,\alpha ,\beta )$ represents the output Q-value of the Q-network.

#### 3.3.1. Target network soft update.

Traditional Deep Q-Network often use hard updates, where the target network is updated once every stable C steps. This paper proposes an alternative soft update idea to ensure that the target network is updated in every iteration, which is equivalent to a network update interval of 1. Soft update uses a convex combination of the current network parameters and the target network parameters to update the network, i.e., the parameters $w\prime_{i}$ of the target network will be updated using the current network parameters $w_{i}$ according to Eq. (25).


$$\begin{array}{c} w_{i+1}^{\prime }=w_{i}^{\prime }+\varepsilon (w_{i}-w_{i}^{\prime }),\;where\;0<\varepsilon \ll 1 \end{array}$$ (24)

In this way, the parameters of the target network change slightly, and the calculated target label values y(r,s′) change relatively smoothly. Then, even if the target network is updated in every iteration, the algorithm can maintain a certain degree of stability. The smaller the soft update interval factor, the more stable the algorithm is. An appropriate soft update interval factor can make the DQN algorithm training both stable and fast.

#### 3.3.2 Dynamic epsilon-greedy decay method.

In the D3QN algorithm presented in this paper, a dynamic greedy strategy is employed [32]. During the training phase, traditional reinforcement learning algorithms use a fixed greedy factor ε, which select actions randomly with probability ε and choose the best action with probability 1−ε. If ε is too small, the environment will not be sufficiently explored in the early training stages, leading to over-exploitation or insufficient exploration. If ε is too large, even in the later stages of training when the agent has fully explored the environment, there will be a significant increase in the probability of randomly selecting actions instead of choosing the optimal action, which contradicts the principle of finding the optimal policy. Therefore, a dynamic epsilon-greedy method with an exponential decay is used to address this issue, as shown in Eq. (26).


$$\varepsilon =\frac{{\left(t+\delta \right)}^{3}}{x}e^{-\sqrt{t+\delta }}$$ (25)

where t represents the number of training episodes, δ represents the designed offset, and x is a variable that changes according to the environment. This method ensures more exploration at the uncertainty of the environment in the early stages of training, and solve the exploration-exploitation dilemma of deep reinforcement learning.

#### 3.3.3. Algorithm description.

When the agent is ignorant of the environment in the early stages of learning, the DQN chooses the most advantageous action at each iteration, such as the action with the maximum value function. Then, the agent will be able to find a suboptimal policy in a short period of time with high learning efficiency. However, there is always a problem of overestimation. The DQN decouples the selection of the target Q-value action and the calculation of the target Q-value in two steps to eliminate the problem of overestimation.

In this paper, the corresponding problem in the DDQN algorithm are not selected according to the optimal policy, instead of a dynamic greedy strategy based on Eq. (26) is used. For the sample importance selection, the previously experienced replay method is used to extract experience samples from the experience replay unit, increasing the utilization rate of important samples and accelerating the training speed of the neural network. Then, Eq. (24)is used as a soft update to ensure that the target network is updated in every iteration, making the DQN algorithm training both stable and fast. A deep neural network is used to approximate the Q-function instead of traditional tabular value recording, which allows the D3QN algorithm to solve large-scale reinforcement learning problems with complex environmental states. The loss function of the deep neural network is shown in Eq. (23). The gradient backpropagation of the neural network updates all parameters of the Q-network. Finally, a competitive network structure is introduced to optimize the neural network, thereby optimizing the algorithm, as shown in Algorithm 1.

**Algorithm 1.** IDS Algorithm

**1:**  Input: minibatch m, action advantage parameter α, state value parameter β, budget T, update factor ε

**2:**  Initialize replay memory M, $p_{1}=1$, Q-Network parameters θ

**3:**  For t=1 to T:

**4:**   Observe $S_{t}$, and choose $A_{t}\sim \pi_{\theta }(S_{t})$ At (St) with dynamic ε−greedy

**5:**  Store transition $<S_{t},A_{t},R_{t},S_{t+1},done>$ in M with maximal priority $p_{t}=\max_{i<t}p_{i}$

**6:**  update state $S_{t}=S_{t+1}$

if M mod m==0

**7:**  For j=1 to m

**8:**   Sample transition $j\sim P(j)=p_{j}^{\alpha }/\sum_{i}p_{j}^{\alpha }$

**9:**   Compute importance-sampling weight $\omega_{j}=(N\cdot P(j){)}^{-\beta }/\max_{i}\omega_{i}$

**10:**  Calculate the current target Q-value $y_{i}={\hat{R}}_{j}+\gamma Q_{t\mathrm{\backslash arg}et}(S_{j},\mathrm{\backslash arg}\max_{a}Q(S_{j},a;\theta ,\alpha ,\beta );\theta ,\alpha ,\beta )$

**11:**  Use the mean square error loss function to backpropagate to update the Q-Network parameters

**12:**  Recalculate TD-error $\delta_{j}={\hat{y}}_{j}-Q(S_{j-1},A_{j-1};\theta ,\alpha ,\beta )$

**13:**  Update transition priority $p_{j}\leftarrow \left|\delta_{j}\right|$

**14:**  Soft update target network $\begin{array}{c} \theta_{t+1}^{\prime }=\theta_{t}^{\prime }+\varepsilon (\theta_{t}-\theta_{t}^{\prime }) \end{array}$

**15:**  end for

**16:** end for

**Fig 5:**
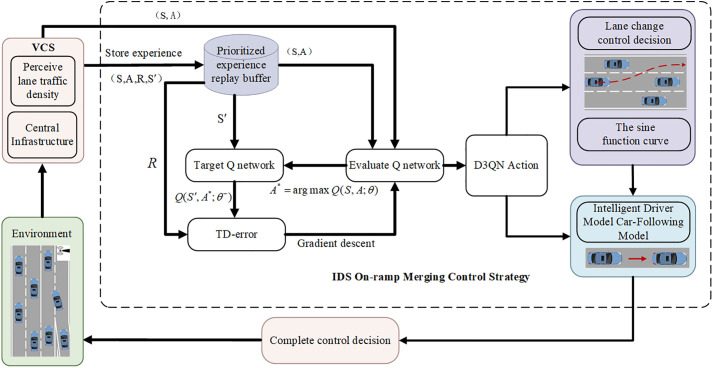
Complete Architecture Combining VCS and IDS.

The architecture that integrates the vehicle coordination system and IDS on-ramp merging strategy proposed in this paper is shown in **Fig 5**. Agents acquire all vehicle information from the road network environment through VCS, and the IDS lane-changing strategy makes corresponding actions. If maintaining the original lane, the IDM car-following model is used directly; otherwise, a lane change is initiated, with the trajectory based on a sine function curve. Ultimately, a complete control strategy is formed and executed on the ego vehicle.

## 4. Experiments and results

### 4.1. Experimental setup

**Fig 6:**
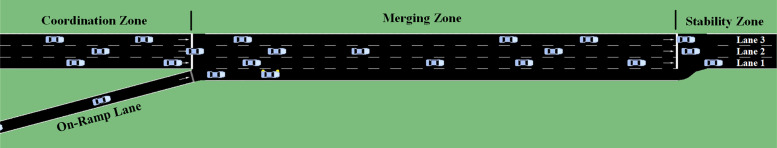
SUMO On-Ramp Traffic Scenario.

The simulation environment in this study is based on the Simulation of Urban Mobility (SUMO) driving simulator [33], as shown in **Fig 1** and **Fig 6**, the mainline consists of three lanes, with a coordination area of 400 meters, a merging area of 100 meters, and a stabilization area of 100 meters. CHVs follow the Intelligent Driver Model (IDM), capable of basic car-following and collision avoidance [34]. The initial speed range of the merging vehicles is [5,25] m/s. When merging into the mainline, vehicles must travel safely for another 100 meters in the stabilization area to complete the task. The speed limit on the mainline is 30 m/s. New vehicles entering the scene are assigned vehicle types of CAV or CHV using a random seed. Traffic flow of low density is 800(veh/lane/h), medium density is 1000(veh/lane/h), and high density is 1200 (veh/lane/h).

The experimental model has a three-layer neural network structure. The number of neurons in the input layer is the dimension of the environmental state. The number of neurons in the output layer is set to the dimension of environmental actions for all models. Rectified Linear Unit (ReLU) is used as the activation function. To facilitate experimental comparison, the hyperparameters of the comparative algorithms and the proposed algorithms are the same, and the hyperparameter settings for the reinforcement learning algorithm are shown in Table 1.

**Table 1:** Algorithm Parameter Setting.

Hyperparameters	Value
Discount factor	0.99
Batch size	256
Memory capacity	38650
Learning rate	1e-5
Priority weight ratio	0.6
Sampling weight	0.4
Optimizer type	Adam

### 4.2. Performance of different methods

To evaluate the performance of the proposed VCS + IDS strategy, comparative experiments were conducted with methods D3QN, DDQN, and VCS+DDQN. The success of vehicles merging into the main road and reaching their destination was assessed. In the experiments, each simulation lasted for a time period of 600 seconds, and each method was iterated for more than 6000 episodes, with data recorded every 20 episodes to verify their performance.

The penetration rate of CAVs was referenced from paper [20], which provided average speed, percentage difference in speed, and corresponding merging area and main road traffic flow under different CAV penetration rates. The average speed increased with the increase of penetration rate. When the CAV penetration rate is less than 60%, the impact on the average speed of the main road and merging speed is small, while the impact is significant when the CAV penetration rate is high (about 60%). Therefore, the CAV penetration rate in this experiment is 60%.

For comparison, three density of vehicle flow (800, 1000, 1200 veh/lane/h) and three demand splits (80−20, 65−35, 50−50) were predefined. Please note that this is the number of vehicles per lane per hour, and the specified demand split is to divide the total capacity. For example, when the traffic density is 800 (veh/lane/h) and the demand split is 50−50, the actual total traffic density of the main road lane and on-ramp lane is 3200 (veh/lane/h), the traffic volume of the on-ramp lane is 1600 (veh/lane/h), and the remaining three main road lanes have a total traffic volume of 1600 vehicles, with an average traffic volume of about 533 (veh/lane/h) per lane.

Let the traffic volumes of each downstream lane in the merging area be denoted as q1, q2 and q3. Then, define the downstream lane imbalance factor u as follows $u=\max(q_{1},q_{2},q_{3})/\min(q_{1},q_{2},q_{3})$, which is always greater than or equal to 1. A larger imbalance factor may cause traffic chaos downstream of the merging area; therefore, we choose a relatively smaller imbalance factor.

**Fig 7:**
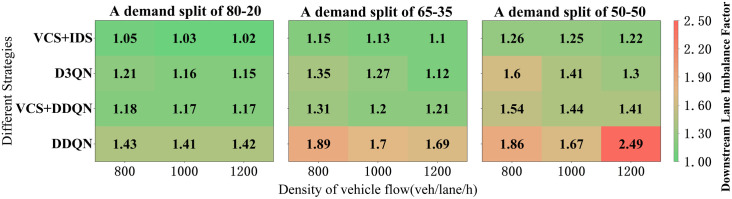
Simulation Results of Different Demand Split.

The simulation results for different assessment scenarios are shown in **Fig 7**, where each table contains a scenario with a specific demand split. In terms of the imbalance factor for downstream lanes, DDQN performs much worse than VCS+DDQN because vehicles in the DDQN strategy cannot comprehensively obtain information about surrounding vehicles, indirectly affecting their driving strategies. The performance of DDQN and D3QN is always lower than when VCS is available, indicating that VCS helps balance traffic volumes across different lanes. DDQN and D3QN perform poorly in complex scenarios and cannot effectively cope with the challenges brought by increased traffic density and changes in demand splits.

VCS + IDS consistently outperforms other scenarios under three different demand splits, especially when the demand split is 50−50. Due to the increased traffic volume from the ramp, the impact on the main road lanes is increased, forcing an increase in the imbalance factor for all strategies. However, VCS + IDS remains relatively stable. Despite the increasing traffic volume requirements, the imbalance factor tends to remain relatively stable, with fluctuations not exceeding 0.03, ensuring a better balance of traffic volumes between lanes, maximizing the utilization of each lane, and demonstrating its high stability in complex traffic environments. Balancing the demand split (from 80−20–50−50) increases the complexity of traffic flow distribution, leading to an increase in the imbalance factor. However, VCS + IDS can still maintain a low imbalance factor value, indicating its significant advantage in balancing traffic flow and reducing traffic chaos.

Overall, VCS + IDS demonstrates stronger robustness and optimization capabilities under high-density and complex traffic conditions through intelligent strategies and coordination mechanisms. It is more effective when the merging area becomes congested, generating better downstream traffic.

**Fig 8:**
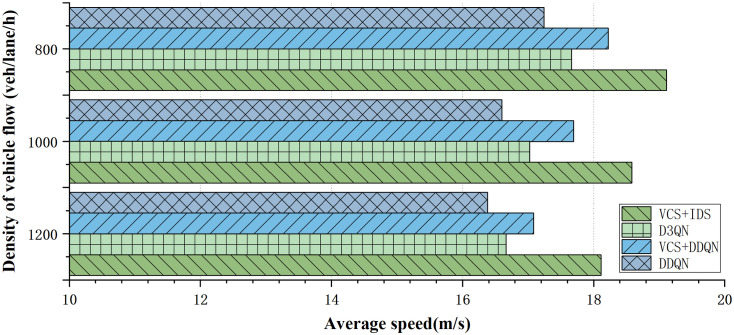
Average Traffic Speed under Different Strategies.

This article presents the average traffic speed under different strategies for completing tasks, as shown in **Fig 8**. VCS + IDS achieved the maximum average speed under various density of vehicle flow. At a traffic density of 800 (veh/lane/h), the speed reached 19.11 m/s, significantly improving overall traffic efficiency by 11.36%, 10.85%, 4.88%, and 8.21% compared to DDQN, VCS+DDQN, and D3QN, respectively. At a traffic density of 1000 (veh/lane/h), the speed can also reach 18.11 m/s, enhancing the average speed by 12.41%, 10.56%, 6.03%, and 8.70% compared to DDQN, VCS+DDQN, and D3QN under the same traffic density. Traditional DDQN and D3QN methods showed limited improvement in traffic efficiency. The integration of VCS+DDQN methods, however, resulted in increased average speeds, with improvements of 5.68%, 6.57%, and 4.27% at different density of vehicle flow compared to DDQN, with the most significant enhancement of 6.57% at 1000 (veh/lane/h). The results indicate that the proposed VCS and IDS can enhance traditional DRL algorithms, as VCS provides strategies that further consider the most appropriate timing for lane changes, thereby reducing congestion rates.

From Table 2, it can be observed that the frequent lane changes in DDQN are also one of the reasons for the low overall traffic efficiency. As the number of vehicles per hour increases, i.e., as traffic density increases, the role of lane changing in improving traffic efficiency becomes more significant. DDQN and IDS equipped with VCS reduced the frequency of lane changes by 33.66% and 69.30% respectively under a traffic flow of 800 (veh/lane/h), by 35.33% and 73.89% under a traffic flow of 1000 (veh/lane/h), and by 47.20% and 76.98% under a traffic flow of 1200 (veh/lane/h). The results indicate that in congested traffic conditions, interactions between vehicles become more frequent. VCS + IDS, when implementing lane change strategies, takes into account the traffic volume of surrounding lanes, significantly improving overall efficiency.

**Table 2:** Lane change frequency for different methods (times/(veh*km)).

Strategies	Density of vehicle flow(veh/lane/h)		
800	1000	1200
DDQN	5.51	5.01	5.72
VCS+DDQN	3.49	3.24	3.02
D3QN	4.56	5.17	5.69
**VCS + IDS**	**1.40**	**1.35**	**1.31**

As shown in Table 3, the collision rates under different strategies are presented. The results indicate that VCS + IDS achieved lower and relatively stable collision rates across various traffic densities, with the lowest being 0.69%, enhancing the safety of lane-changing behavior. With the increase in traffic density, the collision rates for traditional D3QN and DDQN gradually increased: by 0.87% and 1.04% respectively under a traffic flow of 800 (veh/lane/h), by 0.32% and 1.97% respectively under a traffic flow of 1000 (veh/lane/h), and by 1.03% and 3.44% respectively under a traffic flow of 1200 (veh/lane/h). These results clearly demonstrate that VCS + IDS achieved the best performance in the on-ramp merging scenario with mixed traffic on multiple lanes, proving the effectiveness of the proposed VCS and IDS methods, ensuring the driving safety of the mainline and merging vehicles on the road.

**Table 3:** Collision rate for different strategies (%).

Strategies	Density of vehicle flow(veh/lane/h)		
800	1000	1200
DDQN	2.49	3.35	4.01
VCS + DDQN	1.62	3.03	2.98
D3QN	1.81	2.66	4.16
**VCS + IDS**	**0.77**	**0.69**	**0.72**

**Fig 9:**
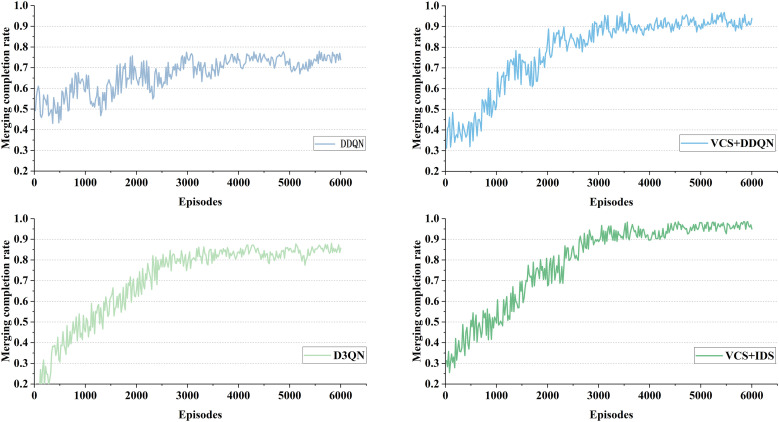
Merging completion rate for different methods at 1200(veh/lane/h).

As shown in Table 4 and Fig 9, the completion rate of on-ramp merging under different traffic densities is presented. DDQN and IDS equipped with VCS improved the completion rate by 11.13% and 6.42% respectively under a traffic flow of 800 (veh/lane/h), by 9.1% and 5.95% respectively under a traffic flow of 1000 (veh/lane/h), and by 19.45% and 11.08% respectively under a traffic flow of 1200 (veh/lane/h). From **Fig 9**, it can be observed that the merging rate of DDQN at the on-ramp is generally moderate, with no significant increasing trend as the number of training episodes increases. In contrast, for DDQN and IDS with VCS, the completion rate is initially low, but it gradually increases as the number of training episodes increases, eventually stabilizing around 4000 episodes.

**Table 4:** On-ramp merging completion rate for different methods (%).

Strategies	800(veh/(lane*h))	1000(veh/(lane*h))	1200(veh/(lane*h))
On-ramp merging completion rate	On-ramp merging completion rate	On-ramp merging completion rate
DDQN	86.45	88.58	76.56
VCS + DDQN	97.58	97.68	96.01
D3QN	92.89	93.07	87.54
**VCS + IDS**	**99.31**	**99.02**	**98.62**

**Fig 10:**
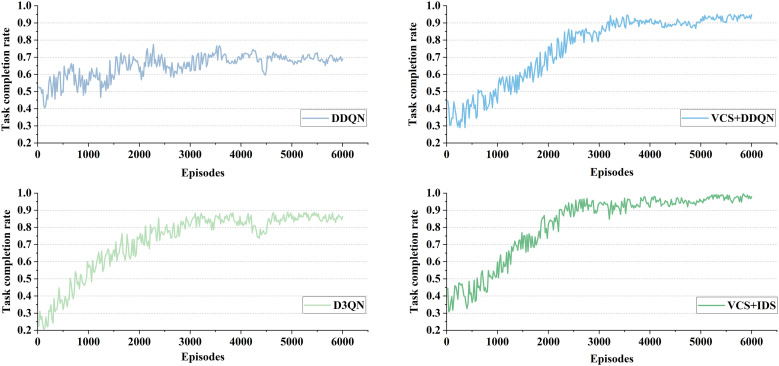
Task completion rate for different methods at 1200(veh/lane/h).

As shown in Table 5 and Fig 10, DDQN and D3QN did not perform very well in terms of task completion rate under different traffic densities. One possible reason is that traditional DRL struggles with multi-lane scenarios due to the complexity of environmental factors, which agents cannot effectively process, leading to increased lane changes and collisions, thereby indirectly affecting the task completion rate. In contrast, DDQN and IDS equipped with VCS improved the completion rate by 11.64% and 8.47% respectively under a traffic flow of 800 (veh/lane/h), by 13.37% and 10.41% respectively under a traffic flow of 1000 (veh/lane/h), and by 24.37% and 10.79% respectively under a traffic flow of 1200 (veh/lane/h). This further demonstrates the effectiveness of VCS and IDS. These results indicate that the proposed methods are applicable to different scenarios and traffic densities, exhibiting better generalization and robustness.

**Table 5:** Task completion rate for different methods (%).

Strategies	800(veh/(lane*h))	1000(veh/(lane*h))	1200(veh/(lane*h))
Task completion rate	Task completion rate	Task completion rate
DDQN	85.32	82.65	70.66
VCS+DDQN	96.96	96.02	95.03
D3QN	90.54	88.35	87.32
**VCS + IDS**	**99.01**	**98.76**	**98.11**

## 5. Conclusion

This paper proposes an improved D3QN on-ramp merging strategy combined with sine functions for multi-lane scenarios. The sine function curve is used to determine the lane-changing trajectory, and the longitudinal control employs the IDM following model. The study divides the road section into three areas: the coordination area, the merging area, and the stabilization area. In the coordination area, VCS (Vehicle Coordination System) is established to coordinate the traffic density between different lanes, ensuring that there is enough space in the merging area for vehicles from the ramp to merge into the main road. A vehicle must safely exit the stabilization area to complete the task. The VCS + IDS method is proposed, utilizing the improved D3QN algorithm, and incorporating safety, efficiency, lane-changing, and task completion rewards in the reward function to ensure the safe driving of vehicles. To validate the effectiveness of the method, simulation experiments were conducted using the SUMO driving simulator platform. Comparative experimental results show that under a traffic flow of 1200 (veh/lane/h), the on-ramp merging completion rate of VCS + IDS is 98.62%, and the task completion rate is 98.11%, which are 11.08% and 10.79% higher than those of traditional D3QN, respectively, showing a significant advantage. It not only improves safety but also enhances the overall traffic efficiency of the road section.

In future research, the impact of driver driving styles on ramp merging can be considered to expand the scope of application of the methods presented in this paper. Additionally, issues such as network environment latency and continuous packet loss in communication are worth further exploration.
